# Cat Respiratory Nematodes: Current Knowledge, Novel Data and Warranted Studies on Clinical Features, Treatment and Control

**DOI:** 10.3390/pathogens10040454

**Published:** 2021-04-10

**Authors:** Simone Morelli, Anastasia Diakou, Mariasole Colombo, Angela Di Cesare, Alessandra Barlaam, Dimitris Dimzas, Donato Traversa

**Affiliations:** 1 Faculty of Veterinary Medicine, University of Teramo, 64100 Teramo, Italy; smorelli@unite.it (S.M.); mcolombo@unite.it (M.C.); adicesare@unite.it (A.D.C.); 2 School of Veterinary Medicine, Faculty of Health Sciences, Aristotle University of Thessaloniki, 54124 Thessaloniki, Greece; diakou@vet.auth.gr (A.D.); dimzas@vet.auth.gr (D.D.); 3 Department of Agriculture, Food, Natural Resources and Engineering (DAFNE), University of Foggia, Via Napoli 25, 71121 Foggia, Italy; alessandra.barlaam@unifg.it

**Keywords:** cat, *Aelurostrongylus abstrusus*, *Troglostrongylus brevior*, *Capillaria aerophila*, diagnosis, treatment, prevention

## Abstract

The nematodes *Aelurostrongylus abstrusus*, *Troglostrongylus brevior* and *Capillaria aerophila* are the most important parasites inhabiting the airways of cats. They are receiving growing attention from academia, pharmaceutical companies and veterinarians, and are now considered a primary cause of respiratory diseases in feline clinical practice and parasitology. In the past few years, several studies have been conducted in both natural and experimental settings to increase knowledge, provide new insights and fill gaps on respiratory parasitoses of cats. Awareness and knowledge of clinical scenarios towards appropriate and timely diagnosis and prompt and efficacious treatment options have become a priority to investigate. At the same time, chemopreventative approaches have been evaluated to assess the geographical spreading of these parasites and the rise in the number of clinical cases in cat populations of different countries. Given the intense accumulation of novel data, this review presents and discusses the state of the art and the latest updates on the clinical features, treatment, and control of major respiratory parasitoses of cats. Moreover, food for thought is also provided with the aim of spurring on new studies in the near future.

## 1. Introduction

The most important parasites inhabiting the airways of cats are the three nematodes *Aelurostrongylus abstrusus* (Strongylida, Angiostrongylidae), *Troglostrongylus brevior* (Strongylida, Crenosomatidae), and *Capillaria aerophila* (syn. *Eucoleus aerophilus*) (Trichinellida, Capillariidae). Other rare nematodes affecting feline lungs, *Oslerus rostratus* (Strongylida, Filariodidae), *Troglostrongylus subcrenatus* (Strongylida, Crenosomatidae) and *Angiostrongylus chabaudi* (Strongylida, Angiostrongylidae), are only occasionally described and their actual roles in feline parasitology are questionable [1,2,3]. Thus, they are out of the scope of the present article. 

The metastrongyloids, *A. abstrusus* and *T. brevior*, are parasites of Felidae, inhabiting and alveoli) and the bronchi, respectively. Their indirect life cycles involve terrestrial gastropods as intermediate hosts. First-stage larvae (L1) of both parasites pass from the respiratory system to the intestinal tract through the pharynx and are eliminated into the environment via the feces of the infected animal. In the intermediate hosts (slugs and snails), L1 develop to second (L2) and then third-stage larvae (L3)—i.e., the infective stage for the vertebrate. Cats become infected by ingesting L3, mostly by preying on paratenic hosts, i.e., rodents, birds, and other small animals, and less often by eating slugs or snails [4,5]. There is solid evidence that *T. brevior* is also transmitted vertically, from infected queens to kittens, while alternative ways of infection, i.e., spontaneous elimination of L3 by infected gastropods, have also been proposed [6,7] but never confirmed in natural conditions.

*Capillaria aerophila* has a wider range of hosts as it infects various carnivore species, including felids [5,8]. In adult stages, they live completely or partially embedded underneath the mucosa of bronchi and trachea and, after mating, the females produce eggs that are transferred via the mucus of the airways up to the pharynx, swallowed and passed in the feces of infected animals to the environment, where they mature. Susceptible animals become infected by ingesting mature eggs harboring infective larvae from the environment; a possible role of earthworms, as facultative or paratenic hosts, has also been proposed [4,9,10].

Parasitoses of the respiratory system of cats have come to prominence in veterinary parasitology in recent years due to their expanding distribution, rediscovered clinical significance and unexpected epizootiological and biological features [1]. Classically recognized as the “cat lungworm”, *A. abstrusus* is a cosmopolitan parasite enzootic in several countries [5,11], while *T. brevior*, a parasite of wild felids, was acknowledged as an agent of verminous bronchopneumonia of domestic cats only recently, and thus far has only been described in Southern and Eastern Europe and the Middle East [1]. In general *A. abstrusus* is more prevalent than *T. brevior.* However, in some cases, such as in Mykonos, an island of Greece, Cyprus, Israel and Apennine regions of Italy, *T. brevior* has been found in higher percentages than *A. abstrusus* [12,13,14,15]. *Capillaria aerophila* also has a worldwide distribution and usually follows *A. abstrusus* and *T. brevior* in terms of infection prevalence in cat populations of the northern hemisphere [4,16,17,18]. 

Specific epizootiological parameters (lifestyle, habitat and age of cats) are risk factors for these parasitoses. As expected, cats with outdoor access are more prone to lungworm infections due to increased opportunities of paratenic host predation [2,12,19]. Cats living in sympatry with wildlife that may act as natural reservoirs of *C. aerophila* (wild carnivores such as foxes) or *T. brevior* (the European wildcat, *Felis silvestris*) are at higher risk of infection by these parasites [1,5,8,20]. Kittens and cats less than one year of age are more commonly infected with *T. brevior* [2,12]. 

For a long time, respiratory nematodes of domestic cats have been underestimated, because (i) the pathogenic ability of *A. abstrusus* has been often overlooked, (ii) *T. brevior* was practically unknown until a decade ago and (iii) *C. aerophila* more often affects wild carnivores. Nonetheless, recent studies have proven that nematodes inhabiting the airways of domestic cats have major roles in feline clinical medicine and they represent a primary cause of respiratory diseases. In the past few years, single reports and case series have provided novel and more comprehensive information on the clinical relevance of feline lungworms towards appropriate clinical workup and reliable diagnosis through conventional and innovative methods. Until a decade ago, practically no anthelmintics were use as treatments for felid lungworms, with very few exceptions. The increasing importance of these parasitoses has spurred a series of field, clinical and experimental studies and research and, to date, a number of molecules and veterinary products have been tested and/or licensed, for treatment and/or chemoprevention. 

The amount of novel data recently generated has offered insights on feline lungworms and have unraveled previously unknown aspects of the associated diseases, especially in terms of clinical aspects and control methods. In consideration of the growing interest in nematodes affecting the respiratory system of domestic cats and the quick accumulation of new information, the present article aims at reviewing and discussing the novel knowledge and latest updates on the clinical features, treatment, and control of these parasitoses, with a focus on new perspectives which have been introduced.

## 2. Clinical Knowledge

The clinical picture caused by feline lungworms is nonspecific and may overlap with those of other feline pathological conditions, e.g., bacterial and mycotic infections, airway foreign bodies, bronchial disease/asthma and lung cancer, thus challenging suspicion for diagnosis [4]. Although *A. abstrusus, T. brevior* and *C. aerophila* adults have different localization (i.e., alveoli, alveolar ducts and terminal bronchioles for the former, bronchi and bronchioles for the second and bronchi and trachea for the latter), both upper and lower respiratory tract clinical signs occur during these parasitoses. Radiographic alterations may be similar to each other, although most knowledge derives from clinical cases of aelurostrongylosis and some gaps have yet to be filled to understand the typical changes (if any) in the infections caused by *T. brevior* and *C. aerophila*. Other imaging methods, e.g., echocardiography, have recently proved useful to evaluate clinical consequences that are often overlooked, such as the potential involvement of heart and pulmonary vessels in lungworm infections. At the same time, more advanced methods, such as Computed Tomography (CT) scanning, represent a potential innovative approach to further investigate the clinical impact of these nematodes on the cardiopulmonary systems of infected cats.

### 2.1. Clinical Signs 

*Aelurostrongylus abstrusus* causes a verminous pneumonia with clinical presentations of varying severity, depending upon worm burden, health status, immune response and age of the infected cat [21,22]. Cat aelurostrongylosis can vary from subclinical and self-limiting signs to severe and potentially life-threatening disease, though most animals present mild to moderate respiratory distress, which first appear after about one month postinfection [4,23,24].

Although nasal cavities are not involved in the L1 migration, upper respiratory tract signs, e.g., sneezing and nasal discharge, occur as a consequence of the inflammation due to the larval transit in the pharynx during swallowing and/or dislocation of bronchial mucous. This pathogenetic mechanism has been hypothesized for *T. brevior*, but the similarities in the life cycle make this assumption plausible for *A. abstrusus* as well [25]. 

Most signs of aelurostrongylosis come from the lower respiratory tract, the most common being dry or productive cough, dyspnea, tachypnea, abdominal, labored and open-mouth breathing [26,27]. End-respiratory crackles and wheezing are also detectable in lung auscultation [27]. Nonspecific and generalized signs such as lethargy, weight loss, anorexia and fever are fairly frequent [23,27]. Severe infections can result in bronchopneumonia complicated by bacterial superinfections, pleural effusion or pneumothorax, and lead to respiratory failure with cyanotic mucosae, respiratory acidosis and death [28,29,30,31].

The inflammation triggered by parasite stages is potentially able to cause secondary pulmonary hypertension (PH). Specifically, reversible PH, right-sided cardiomegaly, pulmonary artery dilatation and systolic tricuspid regurgitation secondary to *A. abstrusus* were first described in a kitten with dyspnea, end-respiratory crackles and heart murmur [32]. Recently, a case of fatal bronchopneumonia in a 6-month-old kitten due to *A. abstrusus* with irreversible PH and right-sided congestive heart failure that resulted in its death has been described [29]. The kitten showed a progressive cough, worsening dyspnea, ascites and pleural effusion [29]. More details on these last two cases are reported in Section 2.3. 

Although immunocompromised cats (e.g., those with concomitant retroviral coinfection) have been traditionally considered for a long time to be at more risk of developing severe clinical signs [24,33], a recent study suggests that the concomitant presence of FIV in cats infected by *A. abstrusus* has no impact in terms of clinical severity, blood parameters, larval count and radiological findings [34]. This could be explained by the fact that most lesions and clinical signs are secondary to the host inflammatory reaction against eggs, larvae and adults of *A. abstrusus* in the respiratory tract [21]. Therefore, the inflammatory response against parasitic stages in immunocompromised animals could be milder if compared to immunocompetent cats. From a clinical standpoint, this means that FIV-positive cats should not always be expected to have more severe clinical signs in case of concurrent aelurostrongylosis.

Other organs than those of the cardiopulmonary complex can sometimes also be involved in clinical scenarios. A fatal case of a kitten that is a few months old due to severe verminous pneumonia and enteritis has been described. In this case, the small intestinal mucosa of the kitten was invaded by a large number of *A. abstrusus* larvae, though it was unclear if this was due to L1 (aberrant invasion) or L3 (exposure to heavily infected intermediate or paratenic hosts), resulting in life-threatening diarrhea [24]. Analogously, L1 has been detected within the colon glands at the necropsy of two cats infected with *A. abstrusus* [35]. However, only partial information is available on clinical signs of these cats before death. In the first case, only depression and fever were described, thus the detection of the larvae within the colon glands could have been an incidental finding, as they were not associated with inflammatory or degenerative changes. In the second case, the cat was found dead, thus no data on clinical signs are available. However, congested lungs with purulent material in the bronchi, flabby heart wall, marked smooth muscle hypertrophy of the pulmonary artery, abdominal effusion and enlarged and congested liver were detected at necropsy [35]. These findings are consistent with bronchopneumonia and right-sided congestive heart failure and, albeit it is only a hypothesis, *A. abstrusus* could be responsible for the death, as a large group of alveoli were found filled with parasite eggs and larvae. In this latter clinical case, colon glands were dilated and filled with mucus and necrotic cellular debris, and lymphocytes and plasma cells were detected in the *lamina propria* of the intestine [35]. Thus, it could be argued that the cat suffered from gastrointestinal signs before death. Gastrointestinal signs (i.e., vomiting and diarrhea) have been described in a 14-week-old kitten that suffered from pneumothorax and pyothorax due to aelurostrongylosis. In this case, a phoretic action of migrating *A. abstrusus* larvae carrying bacteria from the intestine to the lungs has been hypothesized [36].

Laboratory alterations, i.e., leukocytosis with eosinophilia and mild anemia, occasional lymphocytosis, monocytosis and basophilia are not specific and cannot confirm diagnostic suspicion of aelurostrongylosis [21,23].

Clinical presentations due to *T. brevior* infection are similar, yet this nematode is more pathogenic, especially in kittens and young animals [5]. Troglostrongylosis causes catarrhal bronchitis and interstitial pneumonia, which are highly life-threatening in kittens and cats of ages of less than one year; conversely, young adults and older cats are rarely infected by this nematode and are most often subclinically [4,25,37]. 

*Troglostrongylus brevior* represents a threat to feline health as a result of its possible vertical route of transmission, which does not occur in *A. abstrusus* infection. Indeed, adult parasites obstruct bronchioles and cause extensive pulmonary hemorrhage and lung congestion with oedema and parenchymatous hepatization, thus decreasing the surface of respiratory exchange [4,25]. In the case of untimely diagnosis, the respiratory lesions caused by *T. brevior* can be fatal even in the case of administration of appropriate anthelmintics [4,37]. 

Upper respiratory signs, such as ocular-nasal discharge and sneezing, are often described in troglostrongylosis [12,38]. This underlines the importance of avoiding a misdiagnosis of other upper respiratory tract diseases (URTDs), which are extremely common in kittens and young cats and characterized by overlapping clinical features [25,39]. The most frequent lower respiratory signs are dyspnea, tachypnea and coughing, followed by wheezing and increased vesicular breath sounds detectable upon lung auscultation [40,41,42]. Nonspecific and general clinical signs are hypo- or anorexia, lethargy, hyper- or hypothermia, dehydration and poor body condition [12,25].

*Troglostrongylus brevior* causes indirect damages to the heart and pulmonary vessels and may induce an irreversible and persisting PH [41]. This was first described in a kitten which was monitored for three months post-treatment. Although no long-term data are available for this clinical case, it cannot be excluded that PH might persist for a long time or throughout the life of animals once infected with *T. brevior*. Therefore, prompt cardiovascular targeted diagnostics should be performed early with clinical cases of feline respiratory disease in order to prevent later morbidity [29,32,41] and in enzootic areas, *A. abstrusus* and *T. brevior* should be included in the differential diagnosis in cats with heart disease and associated pulmonary hypertension and a compatible anamnesis (e.g., outdoor lifestyle, preying).

As for aelurostrongylosis, hematobiochemical findings in cats with *T. brevior* are nonspecific [25,26]. Leukocytosis is a common alteration, occasionally associated with mild anemia, neutrophilia and monocytosis. Eosinophilia, a common finding in parasitic diseases, is only seldomly present in cats infected by *T. brevior* [25,26,40,41]. 

In most infected cats, *C. aerophila* causes chronic bronchitis that ranges from mild to severe respiratory manifestations. However, on occasion, a fatal outcome may result from diseased animals [8,43]. General distress, dry or productive cough, sneezing with nasal discharge, dyspnea and tachypnea are the most frequent clinical signs [4,44,45]. Common findings on lung auscultation are increased respiratory sounds, end-inspiration crackling and wheezing [44]. Heart murmurs have been reported in some cats, although correlation of this condition with the infection is unclear [8]. Pneumothorax, pleural effusion, interstitial emphysema, lung edema and secondary bacterial infections can also occur [8,44,45]. In these cases, feline pulmonary capillariosis can be life-threatening, regardless of the administration of appropriate anthelmintic and supplementary supportive treatment [8]. As for aelurostrongylosis and troglostrongylosis, laboratory findings are of limited interest. Leukocytosis accompanied with eosinophilia and monocytosis are frequently described, as in many other feline respiratory diseases [8,46]. 

Mixed infections with two or more lungworm species occur frequently in enzootic areas, often resulting in more severe clinical pictures than in monospecific infections [47]. Therefore, when referred with respiratory signs, cats should be examined with appropriate laboratory techniques, i.e., Baermann’s method for *A. abstrusus* and *T. brevior* and flotation for *C. aerophila*, to individuate mixed infections requiring focused parasiticide administrations.

### 2.2. Radiographic Findings

Thoracic radiography is usually the first diagnostic investigation when lower airway and pulmonary parenchymal disorders are suspected. The most frequent abnormalities detected on thoracic radiographs in cats infected naturally [8,41,47,48,49] or experimentally [50] by respiratory nematodes vary from bronchial, nodular, and unstructured interstitial patterns with multifocal distributions, to generalized alveolar patterns in early stages and severe cases. 

Despite the fact that it is generally not safe to attribute specific alterations to a given lungworm species, some species-specific characteristics may be found. Additionally, the correlation between presence and severity of clinical score and radiographic changes is only partial, as many infected cats show radiographic changes without evident clinical signs [33,47,48,51,52], while cats that show clinical signs most often also have evident radiographic abnormalities [26,53,54,55]. In addition, the severity of radiographic signs is not constantly related to age, sex and the lungworm species involved, although the most severe lesions are associated with mixed infections by *A*. *abstrusus* and *T*. *brevior* or by *T. brevior* alone [25,26,47]. 

Radiographic abnormalities in the case of aelurostrongylosis are not specific, often presenting mixed patterns that overlap with those of other conditions (e.g., feline bronchial disease/asthma, infectious pneumonia) [4,47,56,57].

**Figure 1:**
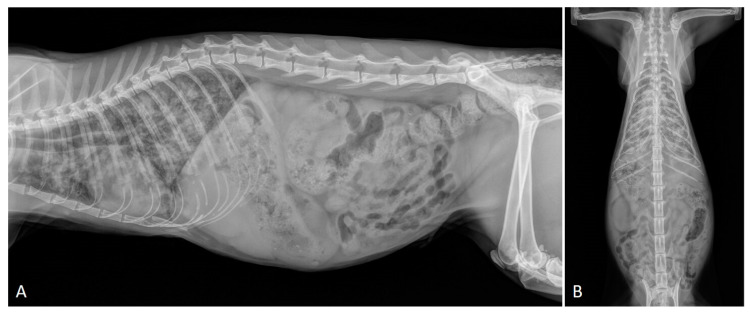
Right lateral (**A**) and dorsoventral radiographs (**B**) of a 3-year-old female domestic shorthair cat displaying evidence of a mixed interstitial-alveolar pattern (courtesy of Chiara Sforzato, Ospedale Veterinario h24 “Abruzzo”, Pescara, Italy).

**Figure 2:**
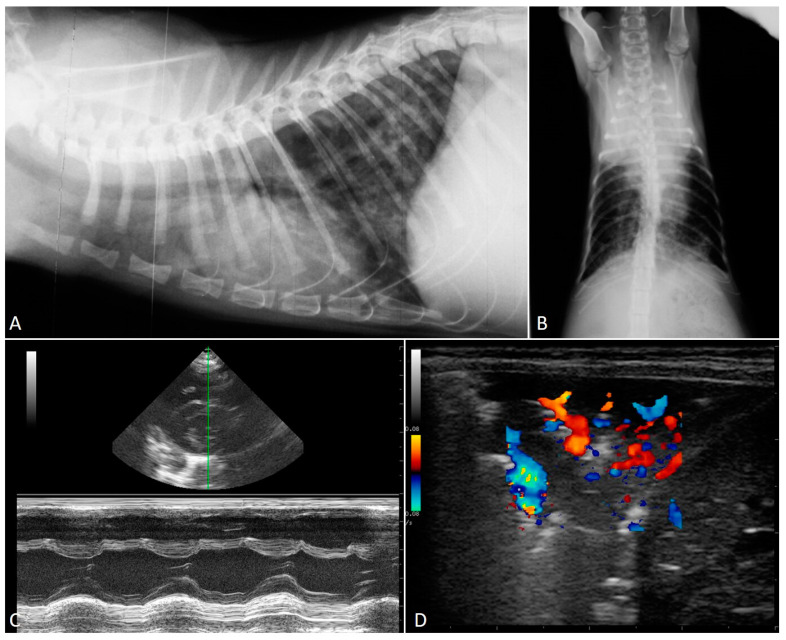
Six-month-old cat infected by *Aelurostrongylus abstrusus*. Right lateral (**A**) and ventro-dorsal (**B**) radiographs showing opacity with alveolar pattern at the cranial lobes and bronchial patterns and hyperinflated areas of the caudal lobes. Right parasternal short axis M-mode echocardiography (**C**) showing dilated right ventricle for pulmonary hypertension. Lung sonography (**D**) conducted at the level of the right dorsal 12th intercostal space showing pulmonary atelectasis of a parenchymal area with visible blood flow on CFM Doppler examination (clinical case by Luigi Venco, Clinica Veterinaria Lago Maggiore, Dormelletto, Novara, Italy, personal data).

Experimental studies have demonstrated that (i) the severity of radiographic findings is associated with the infective dose and chronicity of the infection [50], and (ii) early phases of aelurostrongylosis are radiographically characterized by an alveolar pattern due to mononuclear and eosinophilic exudate surrounding *A. abstrusus* eggs and larvae [50,56]. This alveolar pattern phase usually coincides with the most severe clinical consequences in both experimentally and naturally infected cats—i.e., the presence of overt clinical signs and high-grade lung damage [26,29,56]. The resolution of the alveolar pattern coincides with the emergence of an interstitial pattern which then becomes predominant and is either nodular or diffuse and unstructured (Figure 1), the latter being the most common radiographic feature of clinical aelurostrongylosis [26,47,49]. The presence of a bronchial pattern is also fairly frequent in both experimental and field studies and can be associated with alveolar, interstitial (either nodular or unstructured) and/or vascular patterns [22,32,47,50,56,58] (Figure 2). An enlargement of the cardiac silhouette due to reversible [32] or irreversible [29] PH (see Section 2.1 and Section 2.3) has also been reported. 

**Figure 3:**
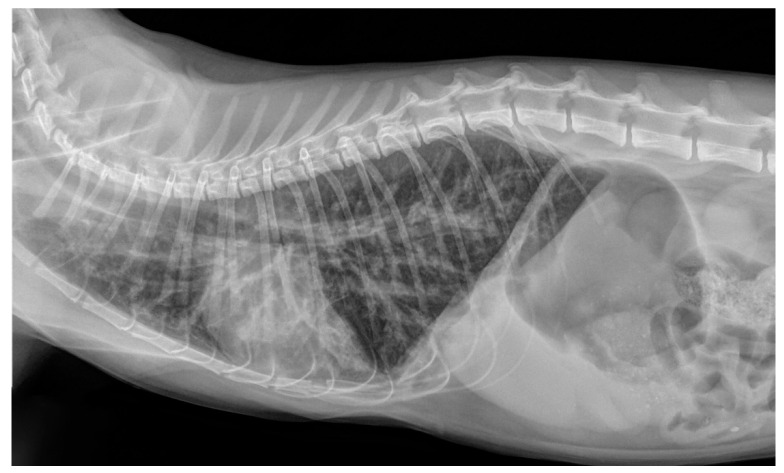
Right lateral radiograph of a six-month-old cat infected with *Troglostrongylus brevior*. Moderate bronchial pattern and pulmonary hyperinflation are observable. The stomach is filled with air, ingested due to severe dyspnea (courtesy of Chiara Sforzato, Ospedale Veterinario h24 “Abruzzo”, Pescara, Italy).

**Figure 4:**
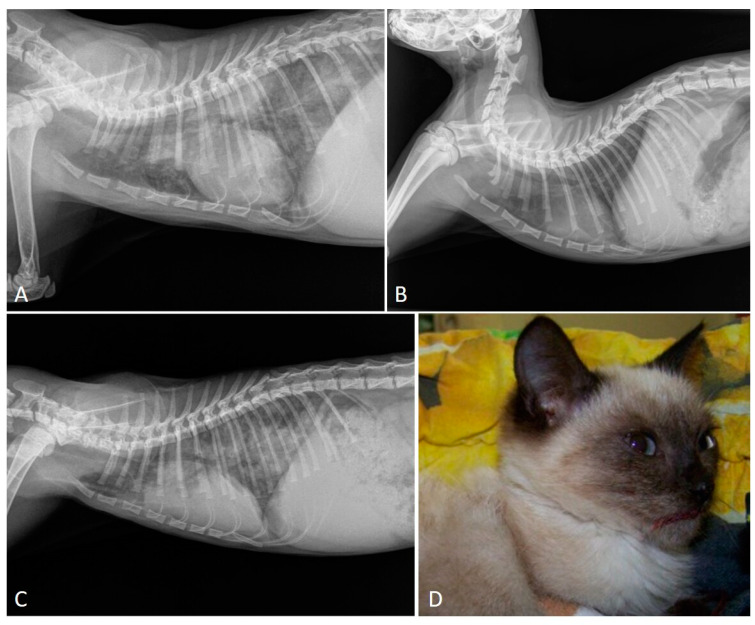
Right lateral radiographs of three 40-day-old kittens from the same litter infected by *Troglostrongylus brevior* and with different degrees of pulmonary damage. Diffuse interstitial and alveolar patterns with enlargement of the cardiac silhouette likely due to right ventricle dilatation (**A**). Absence of pulmonary abnormalities (**B**). Interstitial pattern of the caudal lung lobes and consolidation of the caudal part of the left cranial lung lobe (**C**). Kitten (**D**) of the radiogram (**A**) presented severe dyspnea accompanied by hemoptysis (clinical cases by Luigi Venco, Clinica Veterinaria Lago Maggiore, Dormelletto, Novara, Italy, personal data).

**Figure 5:**
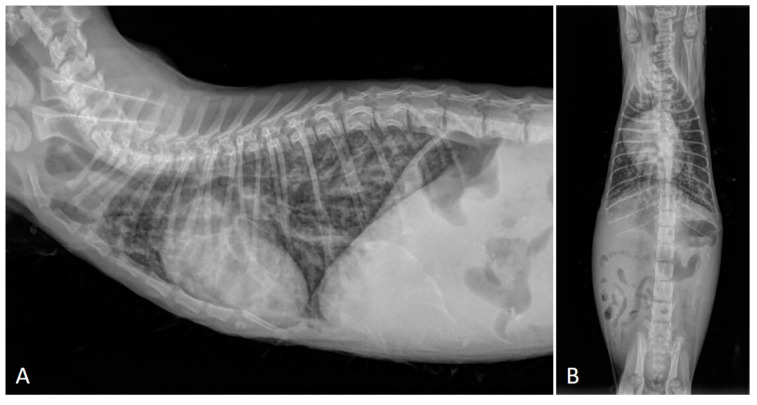
Right lateral (**A**) and ventral-dorsal radiographs (**B**) of a 2-month-old male European shorthair cat infected with *Troglostrongylus brevior* and suffering from pulmonary hypertension. A mixed unstructured interstitial-alveolar pattern is evident along with an enlargement of the cardiac silhouette indicating cardiomegaly (courtesy of Chiara Sforzato, Ospedale Veterinario h24 “Abruzzo”, Pescara, Italy).

Knowledge on the radiographic features of troglostrogylosis is still poor due to the paucity of clinical studies on this disease. The main finding in cats with monospecific *T. brevior* infection is a mild to severe bronchial pattern, associated or not with interstitial and/or alveolar patterns (Figure 3) [26,41,47,59]. The presence of a sole unstructured or nodular interstitial pattern has also been reported [47]. Similar to aelurostrongylosis, a marked alveolar pattern can be present in early and/or severe stages of troglostrongylosis, either alone or mixed with an interstitial and bronchial pattern. For instance, this radiological evidence has been described in 35- and 40-day-old kittens [60,61] (Figure 4) and in a 3-month-old kitten with *T. brevior* monospecific infection [47]. It is interesting to note that thoracic alterations may vary among infected animals belonging to the same litter, which can also present different degrees of clinical signs’ severity [42,61] (Figure 4). As lung damage depends on the number of infectious larvae [50], it is likely that kittens of the same litter acquire a varying number of *T. brevior* larvae vertically transmitted, which results in differing lung parenchymal lesions. PH in course of troglostrongylosis leads to a right-sided cardiomegaly [41], observable using X-ray imaging (Figure 5).

Knowledge of radiographic alterations in feline pulmonary capillariosis is even more fragmentary. Recent studies have indicated that X-ray alterations vary from absent [46] to combinations of bronchial, interstitial and alveolar patterns [8,47]. Two recent series, although on a small number of cases, have also shown that a bronchial pattern is almost invariably present in cats infected with *C. aerophila,* often associated with alveolar and/or interstitial patterns (unstructured or nodular) [8,47]. The presence of an interstitial pattern alone has also been described [47]. 

**Figure 6:**
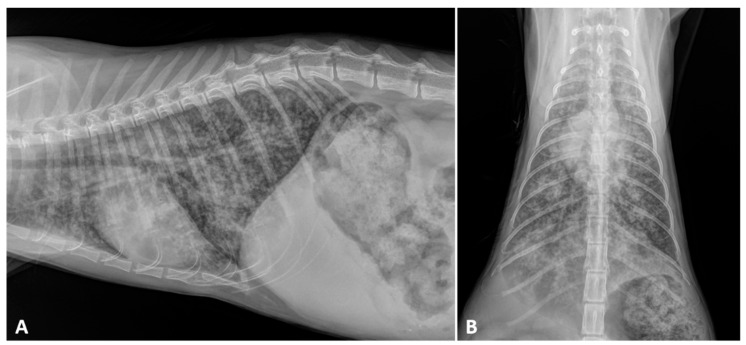
Right lateral (**A**) and ventro-dorsal (**B**) radiographs of a 1-year-old cat coinfected with *Aelurostrongylus abstrusus* and *Troglostrongylus brevior*. Severe and diffuse bronchial-interstitial nodular pattern associated with alveolar pattern associated with alveolar pattern and unilateral pleural effusion are visible (courtesy of Paolo E. Crisi, University Veterinary Teaching Hospital, University of Teramo, Italy).

Precise radiographic data on mixed infections caused by lungworms are poor. A severe bronchial pattern was present in a cat infected with *T. brevior* and *C. aerophila* and a mixed bronchial/unstructured interstitial pattern in cats coinfected by *A. abstrusus* and *C. aerophila* [47]. In a recent study, 6 out 7 cats with mixed infection with *A. abstrusus* and *T. brevior* displayed a mild to severe bronchial pattern sometimes accompanied with unstructured interstitial or nodular, alveolar or vascular patterns or a combination of them (Figure 6) [47]. These findings suggest that a bronchial pattern is very frequent in cats infected with *T. brevior* and/or *C. aerophila* regardless of the presence of other lungworms [47]. In the same study, there was no significant association between the severity of the radiographic alterations and the coinfection by these two metastrongyloids if compared to monospecific infections, in spite of the clinical condition being generally worse in cats with mixed infection [47]. This confirms that a discordance between mere clinical signs and radiographic abnormalities exist in cats infected by respiratory nematodes. Indeed, radiographic changes may be evident before the onset of clinical sings in lungworm infections in both mixed and monospecific infections, underlining the need to perform adequate parasitological diagnostic tests when cats show pulmonary alterations, even if these were accidental findings. 

### 2.3. Echocardiography

The verminous bronchopneumonia triggered by *A. abstrusus* and *T. brevior* may result in cardiac involvement. 

Pathological changes of pulmonary arteries (e.g., disruption of the vascular endothelium, hypertrophy and hyperplasia of the media smooth muscle cells, intima proliferation, lumen occlusion) were described in early experimental infections with *A. abstrusus* [52,62]. More recently, data have been acquired on cardiovascular damages in clinical settings. In a case report, two 3-month-old kittens from the same litter in the Netherlands were referred with dyspnea, sneezing and mucopurulent discharge and diagnosed with *A. abstrusus* infection [32]. As inspiratory crackles and systolic heart murmur were detected at the clinical examination, thorough echocardiography was performed on both animals. One kitten had right-sided cardiomegaly with severe eccentric hypertrophy of the right ventricle, moderate dilated right atrium and a systolic regurgitation from the tricuspid valve. The sudden worsening of the respiratory distress caused the death of this animal. The echocardiography of the second animal revealed a right-sided cardiomegaly, with severe right ventricular dilatation, a moderately enlarged right atrium and the dilatation of the pulmonary artery. Furthermore, a large systolic tricuspid regurgitation, and mild pulmonic valve insufficiency and systolic PH were detected. After two administrations of milbemycine oxime two weeks apart, the kitten recovered parasitologically and clinically and thus the echocardiographic abnormalities and cardiovascular alterations were attributed to the lungworm infection [32]. Although *A. abstrusus* has been considered the aetiological agent, many of the data, i.e., the kittens’ ages, the fatal outcome of one of them, the severity of clinical signs and the fact that they belonged to the same litter, suggest a *T. brevior* infection. In fact, in this report data on morphology and morphometry of the larvae were not published [32]; thus, as knowledge on *T. brevior* occurrence in domestic cats was practically nil at that time, the possibility of a misdiagnosis cannot be ruled out. 

After a clinical study unable to show PH or cardiac abnormalities by echocardiography in fourteen cats with clinical signs due to *A. abstrusus* infection [49], an unequivocal fatal case of aelurostrongylosis with severe PH and congestive heart failure has been described more recently [29]. A six-month-old kitten with cough, progressively worsening dyspnea and heart murmur was subjected to echocardiography, which revealed right atrial and ventricular dilatation and tricuspid regurgitation. These findings, along with mild pericardial, pleural and moderate abdominal effusions, were consistent with a deadly PH and right-sided congestive heart failure [29]. In the latter case the involvement of *A. abstrusus* was ultimately confirmed by the molecular identification of the parasite.

There is one clinical case in which *T. brevior* was demonstrated as a cause of impairment of cardiac functionality [41]. The echocardiographic examination of a four-month-old kitten with dyspnea, open-mouth breathing and heart murmur revealed severe right-sided cardiac enlargement, systolic tricuspid regurgitation and PH. After the administration of an efficacious anthelmintic, the clinical condition improved, although mild dyspnea, increased respiratory sounds and heart murmur were still present up to three months post-treatment. Being still detectable at the follow-up echocardiography, the PH was considered “irreversible”, indicating that it might persist for a long time in cats with extended damages to the lung parenchyma [41].

Different hypotheses could explain the pathogenesis of PH in lungworm-infected cats. The host inflammatory response towards nematode adults, larvae and eggs can induce the hypertrophy of the bronchial musculature, along with the hypertrophy and the hyperplasia of the smooth muscle cells of the pulmonary arteries [32]. The consequent narrowing of diameters of the pulmonary vessels and of the bronchial system promotes the development of PH [29,32]. The onset of PH has also been attributed to a progressive hypoxemia triggered by a decrease in the functional alveolar surface as a result of lung damage. The increasing resistance of the pulmonary vasculature could also be due to the vasoconstriction of the pulmonary vessels activated by mast cells and histamine [29,32]. 

No data on possible indirect cardiovascular effects secondary to *C. aerophila* infections in cats have been published, though a recent study has shown that heart murmur may be present in cats with pulmonary capillariosis [8].

### 2.4. Computed Tomography Scan

CT scan is less commonly used than conventional radiology in the diagnostic evaluation of respiratory diseases of cats, although it is increasingly applied due to several advantages compared with conventional radiology. 

Advanced imaging techniques allow a better characterization of lung infiltrates and pulmonary diseases, including elimination of superimposed anatomy and superior contrast resolution [63]. These features allow the visualization of intrathoracic lesions even when radiographic findings are negative or nonspecific. CT has a high accuracy (i.e., almost double) in characterizing lower airway disease, revealing more lesions, and it can better define poorly delineated bronchial walls and nodular lesions and is more efficient at evaluating lymphadenopathy compared to conventional radiology [49,64,65,66]. 

CT findings have been described for cat aelurostrongylosis either in natural or experimental conditions [49,50,65]. In natural infections the predominant lesions consist of multiple nodules of varying size, widely distributed throughout the lungs associated with interstitial–alveolar infiltrate, multifocal nodular structures, generalized lymphadenopathy, bronchial wall, subpleural thickening and multiple small hyperattenuating areas, widely distributed throughout the lung fields [49,65]. Experimental findings are consistent with data from natural infections and have confirmed that the severity of lesions is correlated with the parasitic burden. Cats with induced aelurostrongylosis show nodules in the lung parenchyma, multifocal thickening of the bronchi and interstitial changes blurring the margins of the outer serosal surface of bronchi and vessels [50]. 

A recent study has ultimately proven the high accuracy of CT analysis in evaluating pulmonary damages caused by lungworms, though in a small cohort of cats—i.e., six infected only by *A. abstrusus*, one only by *T. brevior* and one infected by both [47]. Multilobar involvement of the lungs, with an interstitial ground glass opacity, was observed in all cats. Moreover, (peri-)bronchial changes, together with an alveolar infiltrate that was not observed at thoracic radiographs of the same cats, were recorded in five animals with *A. abstrusus* and in the cat with the mixed infection. Additionally, four cats with aelurostrongylosis displayed multifocal nodular lesions which were undetectable at the radiographic examination. This study provided the only CT analysis of a cat with troglostrogylosis described thus far, a mild interstitial ground glass [47].

No data are available on CT examinations in cats with pulmonary capillariosis.

## 3. Parasitological Diagnosis

Diagnosing respiratory parasitoses in cats is pivotal for a timely and efficacious treatment. In recent years there has been a significant generation of knowledge on conventional and innovative methods to detect *A. abstrusus*, *T. brevior* and *C. aerophila*. Traditional methods rely on the identification of diagnostic stages (L1 for *A. abstrusus* and *T. brevior*, and eggs for *C. aerophila*) in the feces of infected animals. Nonetheless, copromicroscopy may have inherent drawbacks which can be overcome by innovative genetic assays. Very recently, serological methods have also been developed. All these tools are used in studies under research and clinical settings instrumental to diagnosis, treatment and control of these parasites.

### 3.1. Microscopic Methods

**Figure 7:**
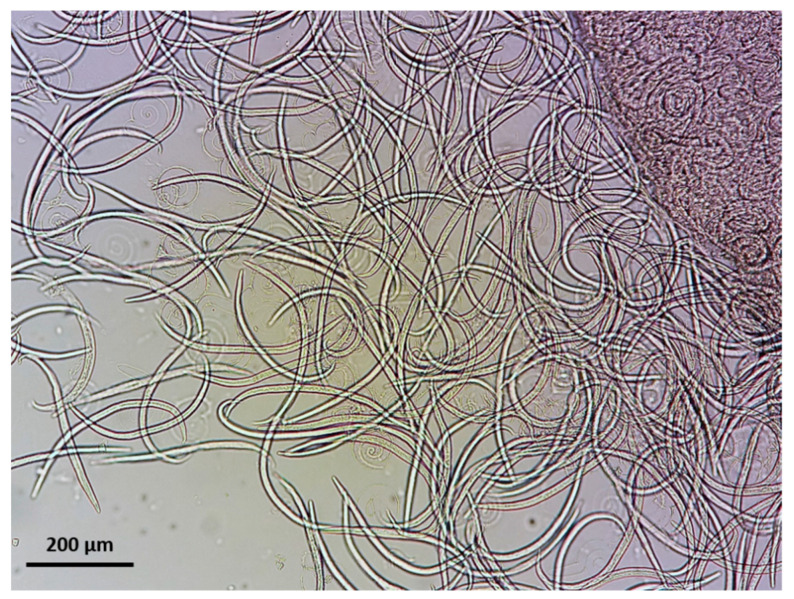
*Aelurostrongylus abstrusus* L1 found in broncho-alveolar lavage of an infected cat.

The diagnostic stages of *A. abstrusus* and *T. brevior* are L1, which are present in the mucus of the airways that normally ascends the trachea and passes to the pharynx [5,67], while coughing facilitates this passage. Consequently, L1 can be found in broncho-alveolar lavage (BAL) (Figure 7), pharyngeal swabs, and respiratory discharge of the infected animals [33,68]. However, feces are the most suitable biological sample for detecting L1 under the microscope. In fact, L1 reaches the environment via the feces after the infected cat swallows the respiratory mucus. It is worth noting that intermittent larval shedding or a very low number of L1 in feces may pose a diagnostic challenge in some cases and cause false negative results [21]. Thus, examination of samples from more defecations (e.g., three consecutive samples) may be required in case of a negative result in suspected clinical presentations.

**Figure 8:**
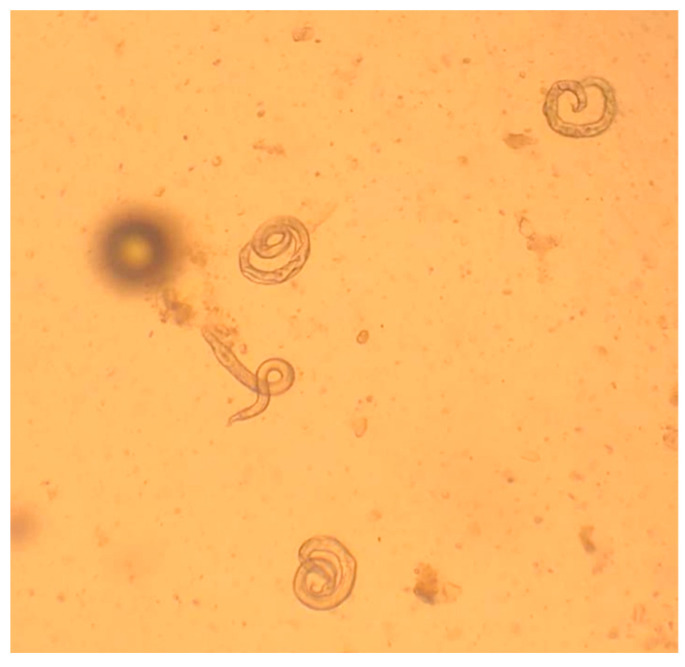
Dehydrated *Aelurostrongylus abstrusus* L1 at a fecal flotation with saturated salt solution, (courtesy of Claudia Segato).

**Figure 9:**
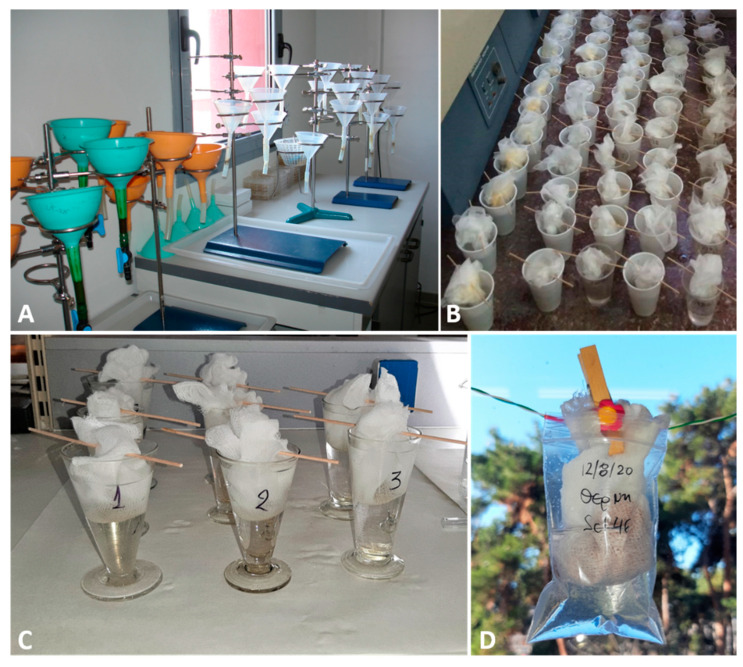
Baermann’s method applied in various conditions. Apparatus settings in a laboratory oriented to lungworm research (**A**), in a veterinary clinic (**B**), in a laboratory with basic equipment (**C**), in the field (**D**).

**Figure 10:**
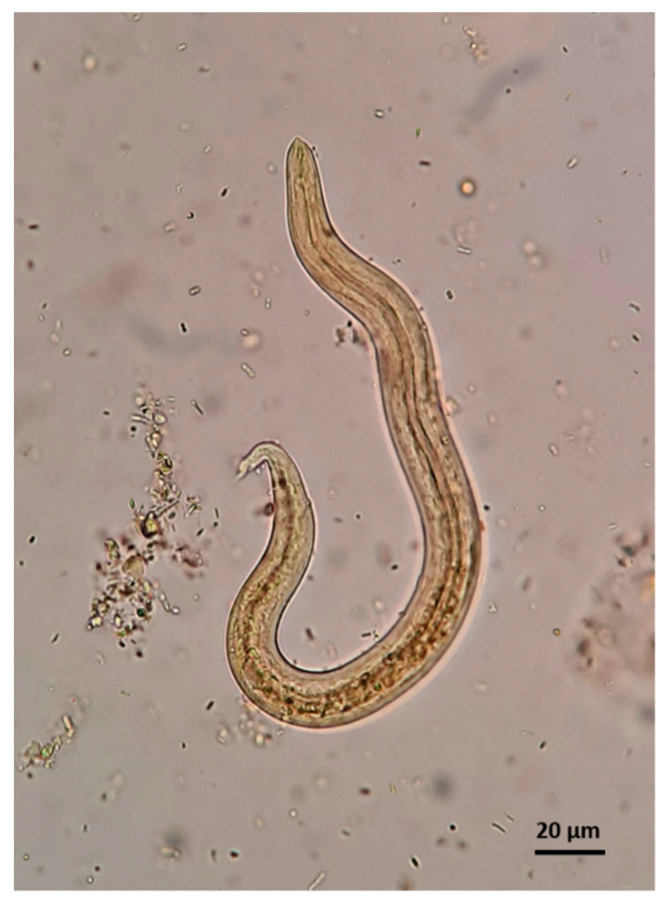
*Troglostrongylus brevior* L1 found in fecal examination by ZnSO_4_ flotation method (lugol stained).

The detection of L1 in feces is feasible by classical parasitological methods such as flotation or sedimentation [69,70], although the gold standard diagnostic technique is Baermann’s test, which presents higher sensitivity and allows detection and identification of L1 [5,69,71]. It should be taken into account that salt solutions may dehydrate the larvae which, in this case, sink, shrink and/or become difficult to identify [11] (Figure 8). Baermann’s test can be conducted with various setting arrangements (Figure 9) depending on either field, clinical or laboratory conditions. With this method, the L1 migrate from feces to the water and can be collected after 12–24 h from the sediment of the liquid in the Baermann’s apparatus. Fresh fecal samples or stored at refrigeration temperatures for a few days are required for the test, so that the larvae remain alive and sufficiently motile. In case of dead larvae (e.g., frozen feces), ZnSO_4_ flotation is the next best option, as larvae retain their morphological features and can be identified (Figure 10). L1 of *T. brevior* are more resistant than those of *A. abstrusus*, being very tolerant to low temperatures and remaining alive and motile even in feces kept frozen for a long time. 

**Figure 11:**
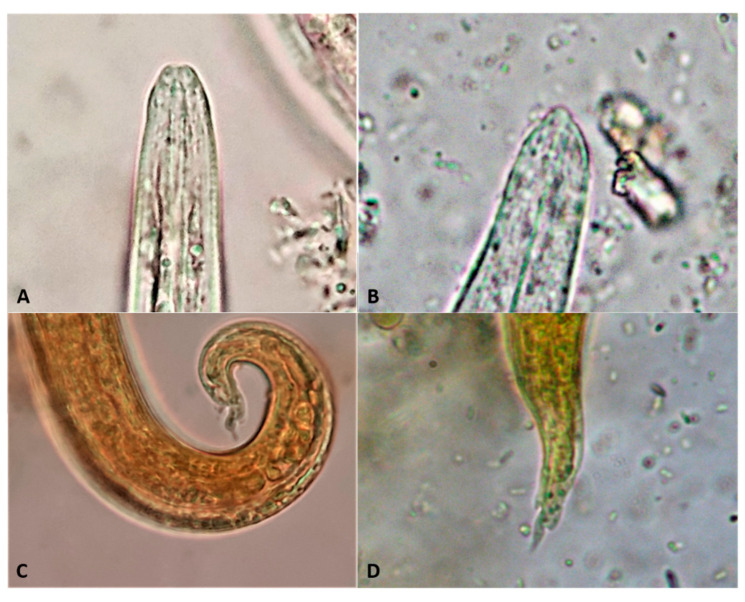
Comparative morphology of anterior (**A**,**B**) and posterior (**C**,**D**) extremities of *Aelurostrongylus abstrusus* (**A**,**C**) and *Troglostrongylus brevior* (**B**,**D**). C and D: lugol stained. x1000 magnification.

Under the microscope, *A. abstrusus* and *T. brevior* L1 may appear similar, though their discrimination is possible based on specific morphometric and morphological characteristics [5,72,73] (Table 1, Figure 11). 

Although *A. abstrusus* and *T. brevior* L1 overlap in length and width, the former is generally longer than *T. brevior* and has a wider range of length measurements (Table 1). The microscopic appraisal of larval extremities allows the differentiation of *A. abstrusus* and *T. brevior* L1. The anterior end of *A. abstrusus* L1 is simple and appears as a plateau with a terminal oral opening. Its characteristic sigmoid posterior end shows a prominent ventral kink, deep dorsal and ventral incisures, and a terminal knob-like ending (Figure 11). Conversely, the characteristic anterior end of *T. brevior* L1 has a subterminal oral opening and a clearly visible apical prominence. Its posterior extremity has a shallow or absent dorsal kink, a deep dorsal and a shallow ventral incisure and a slender terminal appendix (Figure 11).

**Figure 12:**
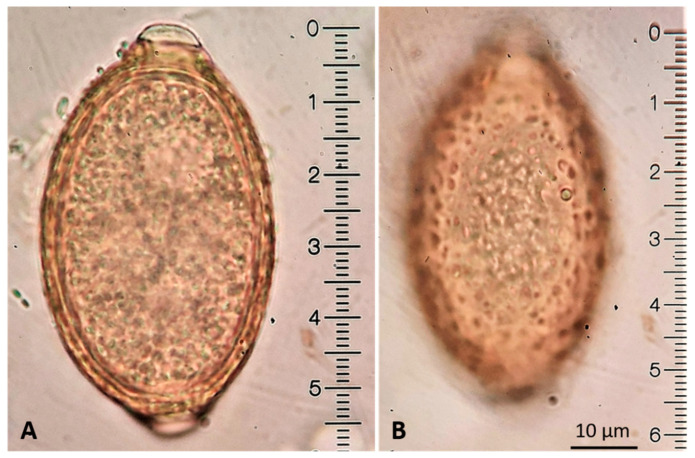
*Capillaria aerophila* egg morphology. Barrel-shaped, yellow egg with asymmetrical polar plugs (**A**). Ridges forming a network of anastomoses of the egg surface (**B**).

*Capillaria aerophila* eggs may be found both by flotation and sedimentation methods [69,71]. Due to their characteristic yellowish color, lemon shape, and bipolar appearance, they may be confused with *Trichuris* eggs. This is a diagnostic problem mainly in canine parasitology because the felid whipworm *Trichuris felis* [75] is extremely rare or even absent in most areas of the world [4]. Nevertheless, the presence of *Trichuris* eggs in cat feces, either due to factual whipworm parasitosis or due to pseudoparasitism (e.g., *Trichuris muris* eggs, in case the cat preyed an infected rodent), should be correctly distinguished from those of *C. aerophila*. There are specific morphometric and morphological differences between *C. aerophila* and *Trichuris* eggs, which are clearly visible under the optical microscope. The egg size allows only the discrimination between *C. aerophila* (64–66 × 32–40 µm) and canine *Trichuris vulpis* (72–94 × 31–42 µm) [76]. However, *T. felis* and *T. muris* eggs have overlapping sizes with *C. aerophila* [75,77], thus in this case other morphological characteristics will support the accurate differentiation. The eggs of *C. aerophila* display a network of anastomosing ridges on the wall surface, asymmetrical polar plugs and absence of a thickening with ringed appearance at the base of the polar plugs (Figure 12). These rings are conversely present in *Trichuris* spp. eggs, that in addition have a smooth egg wall [76]. Differentiation of *C. aerophila* from other capillariid ova present in the feces of cats praying infected birds (pseudoparasitism) [11] is achieved mainly by the observation of the characteristic surface of the eggs [78]. 

### 3.2. Serology 

The drawbacks of microscopic examination, i.e., the discontinuous shedding or absence of L1, the fact that fresh fecal samples are often hard to collect and that experienced personnel are required for the microscopic identification of the larvae, may be surpassed by the application of serological tests. 

Attempts to develop serological tests for aelurostrongylosis reach back to around 50 years ago, when an indirect fluorescent test (IFAT) was developed. This assay, although sensitive and specific, required the use of L3, retrieved from mollusks, as antigens [79]. Similarly, L1 from infected cats have been used as antigen with encouraging results in an IFAT developed some decades later [80]. Both IFATs displayed useful characteristics such as the early diagnosis—i.e., from 3 weeks [79] to 29 days [80] postinfection, and the absence of cross-reactivity with common feline endoparasites [79,80]. However, some important disadvantages—i.e., the need of maintaining infected gastropods or cats and the refined and time-consuming procedures of collecting L3 and L1, respectively, impacted negatively on the validation of these tests as routine procedures. 

More recently, an enzyme-linked immunosorbent assay (ELISA), using a recombinant major sperm protein (MSP) from the bovine lungworm *Dictyocaulus viviparus* as antigen, has been successfully developed [81]. MSP, present in male adult worms, is highly conserved among nematodes and in its recombinant form offers important advantages to serodiagnosis if compared to native antigens due to the potential of a standardized and inexpensive production of stable quality antigen in large amounts [81,82]. This method showed high levels of sensitivity (88.2%) and specificity (90%), providing a useful tool for individual and mass screening diagnosis of lungworm infection [81], as shown in sero-epizootiological studies carried out in Italy, Switzerland and Greece [83,84,85,86]. These studies showed that the infection prevalence obtained by serology is higher compared to values revealed by copromicroscopy both at the animal and study area levels [83,84,85,86]. This discrepancy may be attributed to the intermittent shedding of L1 or to occult infections due to either prepatent phase (i.e., cats seroconvert 2 weeks postinfection (before patency)) or to chronic or repeated infections, where L1 are often not detected in the feces [23,87]. Additionally, antibodies can be detected for a period of 4–6 weeks after the death of adult worms, which may occur spontaneously or as a result of successful anthelmintic treatment [81,84,85]. Consequently, a seropositive sample by this ELISA does not always correspond to a current, active infection, but eventually to a recent, past infection. On the other hand, false negative results in serodiagnosis can occur when the antibody concentration is below the threshold due to recent infection, considering that this method reaches the highest sensitivity 10 weeks post infection or due to concomitant immunosuppressive diseases that suppress antibody production [81].

According to the results obtained in surveys where fecal and serological examinations were applied in parallel in naturally infected cats [84,86], it is unclear if the MSP antigen discriminates *A. abstrusus* from *T. brevior*. In fact, in these studies 3 out of 8 cats in Italy and 5 out of 11 in Greece, which were shedding only *T. brevior* L1, were found to be seropositive by the ELISA. These results may be attributed to (i) a concomitant, occult aelurostrongylosis, (ii) a recent, past infection with *A. abstrusus*, or iii) a true cross-reaction of the anti-*Troglostrongylus* antibodies with MSP [84,86]. Further studies evaluating potential cross-reactions between antibodies against *T. brevior* and MSP antigen would hopefully elucidate the characteristics of this diagnostic method. Additionally, as *T. brevior* belongs to a different family than *A. abstrusus*, a monospecific serological method for *T. brevior* diagnosis using a specific excretory or somatic protein would be of particular value, especially where this nematode is now enzootic and in regions where it is spreading [1]. 

No information on possible cross-reactions of MSP recombinant antigen with anti-*C. aerophila* antibodies is available, although they are unlikely, as this nematode belongs to a different family [81]. 

As neither copromicroscopy nor serology can detect 100% of the infected animals, a combination of these methods is desirable both in clinical (e.g., pre-surgery examinations or investigation of clinical, occult lungworm disease) and in epizootiological mass screening levels. Thus, further studies are needed for the development of commercially available laboratory (kits providing pretreated ELISA wells and accompanying reagents) and in-clinic rapid individual tests for the detection of *A. abstrusus* and *T. brevior*. The latter tests, based on the principle of lateral flow immunochromatography for the detection of circulating antigen or antibodies, specific for each of these two parasites and possibly on the same test cassette, would be very useful and practical for in-clinic use in daily practice. 

### 3.3. Endoscopy and Bronchoalveolar Washing

Bronchoscopy and bronchoalveolar lavage (BAL), though mentioned and described in some studies, do not represent useful diagnostic tools in cats infected by lungworms. 

BAL fluid results in the detection of L1 of *A. abstrusus* in animals with high larval outputs [88] but in general constitutes fewer cases than the fecal Baermann test [68]. 

Bronchial alterations and cytological findings in cats infected by *A. abstrusus* are nonspecific and overlap with those of other feline airway diseases. Therefore, bronchoscopy and BAL do not represent reliable methods for detecting lungworms unless larvae are retrieved. Indeed, fecal examinations remain the first step for diagnosing respiratory nematodes in cats, as bronchoscopy and BAL are invasive techniques that require sedation or general anesthesia (which are hazardous in infected cats) and still may fail to allow a diagnosis in the absence of significant pulmonary tissue involvement, limited numbers of L1s, prepatent infection or poor sample recovery [11,26,68]. 

A partial utility may emerge when these methods are applied to cats with clinical signs in the absence of a respiratory parasitosis suspicion. In fact, some findings (e.g., bronchiectasis) are more frequent in cats infected by *A. abstrusus*, and the detection of hypercellularity with an eosinophilic inflammation suggests potential parasitic infection, though this latter finding is inconsistent and occasional [58,68,88]. In these latter cases, in the absence of larvae at the BAL, further methods (e.g., fecal examinations and/or PCR) are necessary to confirm or exclude a respiratory parasitosis. 

To date, no evidence is available on the use of bronchoscopy and BAL analysis in cats with *T. brevior* and/or *C. aerophila*. Nonetheless, there is no reason to hypothesize they have a higher utility than that described for aelurostrongylosis.

### 3.4. DNA-Based Assays

PCR assays are excellent tools for the genetic discrimination and identification of *A. abstrusus*, *T. brevior* and *C. aerophila* in varying biological samples. These DNA-based protocols amplify specific markers within the ribosomal (rDNA) internal transcribed spacer 2 region (ITS2) for *A. abstrusus* and *T. brevior* and within the gene encoding for the mitochondrial DNA (mtDNA) cytochrome oxidase 1 (cox1) for *C. aerophila*.

The first molecular assay specific for *A. abstrusus* was a two-step semi-nested PCR assessed using a panel of different biological samples from naturally infected cats—i.e., feces, flotation supernatant, Baermann sediment and pharyngeal swabs. The sensitivity and specificity of this tool were 97% and 100%, respectively, and it proved to be able to detect the nematode DNA in copromicroscopically negative samples [89]. Then, the applicability of this PCR to diagnose the infection in animals that tested negative upon conventional fecal examinations was proven in field clinical studies [27]. A similar species-specific PCR proved effective in identifying *T. brevior* in feces and pharyngeal swabs of naturally infected cats, as well as in the case of mixed infections with *A. abstrusus* or *C. aerophila* [42,90]. A refinement of the aforementioned tools was a triplex PCR [91], which is able to detect and differentiate *A. abstrusus*, *T. brevior* and *A. chabaudi*, at the same time—i.e., closely related nematodes affecting pulmonary vessels of wild felids which rarely infect domestic cats [1]. 

The abovementioned molecular tools proved to be powerful in a number of studies, to confirm copromicroscopic diagnoses in clinical cases [29,41,42,58,60], in postmortem evaluations [60,92], in epizootiological and biological surveys [2,14,15,20,40,93,94,95,96] and in the clinical evaluations of anthelmintics [97]. 

Laboratory and field evidence from these studies has suggested that pharyngeal swabs are probably the most useful samples for the molecular diagnosis of aelurostrongylosis and troglostrongylosis. This is based on the sensitivity achieved in various experiments and for practical reasons—i.e., obstacles in collecting adequate cat feces in the field, false negativity due to prepatent and larval migration periods, difficulties in the DNA extraction from fecal samples and possible presence of PCR inhibitors in stool. 

Additionally, a duplex PCR specific for the ITS2 of *A. abstrusus* and *T. brevior* originally developed to discriminate fecal L1 in a single cat with a mixed infection [98], proved to be applicable to the identification of the parasites in a large number of cats in field epidemiological settings [18,99].

A diagnostic assay specific for *C. aerophila* displayed a specificity of 100% and sensitivity of 97–100% using fecal samples from animals scoring positive or negative using conventional methods for *C. aerophila* and other endoparasites, including *A. abstrusus* [96]. This tool proved also useful for phylo-geographic investigations on *C. aerophila* in different host species and geographic regions, leading towards a better epizootiological knowledge of lung capillariosis [100].

## 4. Treatment and Control

### 4.1. Aelurostrongylosis

Until 2015, only a few options were available to treat *A. abstrusus* infections with labelled products, i.e., oral formulations containing fenbendazole in the UK and a spot-on formulation containing moxidectin 1% in New Zealand and Australia, and most protocols were empirical and/or off label. In the past few years, new treatment and control options have been investigated and new data are now available. 

#### 4.1.1. Off Label Treatments 

Ivermectin has been used in single cases, but this molecule has shown inconsistent activity. Although off label ivermectin 400 µg/kg SC has been recommended [101], this molecule in formulations intended for livestock may be toxic, especially in kittens [102]. The administration of two doses of 300 μg/kg SC abamectin, two weeks apart, has been described for treating *A. abstrusus* but, again, this is an off label protocol with a formulation intended for ruminants [103].

Formulations marketed for cats containing selamectin and milbemycin oxime at different dosages and treatment schemes were used against *A. abstrusus* only in single clinical cases or small case series. There is evidence indicating that 18 mg/kg spot-on selamectin reduces clinical signs after a single administration, while a second dose, one month later, may guarantee improvement of respiratory performances and bronchial lesions at the radiographic examinations [104]. Other reports have shown that selamectin was effective in eliminating larvae from the feces of a cat after 30 days [22] and in the clinical recovery and stopping larval shedding in nine out of ten cats infected by *A. abstrusus* [105]. 

Oral milbemycin oxime, which is present in the market in combination with other parasiticides or not, also appeared to be promising. For instance, milbemycine oxime (in a product with praziquantel) given at the dose of 4 mg/kg at two-week intervals was effective in stopping L1 shedding and in the resolution of clinical signs over a period of six weeks [32]. Additionally, a single dose of 2 mg/kg milbemycine oxime was efficacious in stopping larval shedding in a cat with subclinical mixed infection by *A. abstrusus* and *T. brevior* [42]. In this latter study, the same treatment administered to a second cat of the same litter with mixed infection succeeded in stopping *A. abstrusus* larvae shedding, but *T. brevior* larvae shedding continued, and the cat died despite the treatment, with severe coughing and dyspnea persisting after therapy [42]. The different outcome and efficacy of the anthelmintic treatment in this latter study was explained by a different parasitic burden in the same offspring due to the number of *T. brevior* larvae passed from the queen to each kitten of the litter. This study clearly indicated that, though belonging to the same litter, some kittens might harbor a higher number of adult *T. brevior* than others and are at higher risk of severe and deadly lung alterations. This has a potential impact on the efficacy and outcome of parasiticide treatments.

#### 4.1.2. Licensed Products 

To date, formulations containing fenbendazole, emodepside, moxidectin and eprinomectin are used for aelurostrongylosis. 

Fenbendazole is licensed in oral formulations in some countries. Two trials in natural conditions were conducted in the late 2000s to compare the efficacy of fenbendazole against *A. abstrusus* with that of two spot-on solutions, one containing moxidectin 1% (and the neonicotinoid insecticide 10% imidacloprid) and the second containing the cyclooctadepsipeptide 2.1% emodepside (and the cestodicide 8.6% praziquantel) [106,107]. Based on the study data, the fenbendazole oral paste at the dosage of 50 mg/kg *per os* in a 3-day regimen, and the topical solutions containing emodepside and moxidectin in single applications proved to be 99.29%, 99.38%, and 100% efficacious in stopping larval output, respectively [106,107]. Additionally, all cats showing respiratory signs clinically recovered after the anthelmintic treatment [106,107].

Then, results of two experimental trials proved that two applications of the topical parasiticide containing 2.1% emodepside two weeks apart are safe and highly efficacious for the therapy of *A. abstrusus* infection in terms of reducing pulmonary worm counts and fecal larval counts [108]. The product is now licensed for the treatment of aelurostrongylosis in the above regimen. 

After a field study published more than one decade ago [106], the spot-on product containing 1% moxidectin was licensed in extra-EU markets to treat cat aelurostrongylosis. Afterwards, three laboratory studies [109] were carried out to confirm the efficacy of 1% topical moxidectin in the control of aelurostrongylosis by investigating (i) the therapeutic efficacy of up to three monthly treatments against patent *A. abstrusus* infections, (ii) the preventative activity when cats are treated before (10 days) and after (18 days) the infection, and (iii) the preventative activity when cats are treated twice at monthly intervals before the infection. Data generated in this series of studies showed that (i) a single treatment with 1% moxidectin in experimentally infected cats has an efficacy of >88%, (ii) the efficacy rates reach >99% after three treatments in monthly intervals, and (iii) monthly administration is able to kill early larval stages and prevent lung damage and patent aelurostrongylosis [109]. To date, this formulation is licensed in the EU for the treatment of adult *A. abstrusus* with once-a-month administration for three consecutive months, and for the prevention of the disease against L3 and L4 with once-a-month administration. 

The formulation containing 0.4% eprinomectin (and 8.3% praziquantel, 8.3% fipronil and 10% (S)-methoprene) was first evaluated against larval and adult *A. abstrusus* in experimentally infected cats [110]. Its high efficacy, i.e., from 91.6% (fifth larval stage, L5) to >99.9% (adult worms), was shown at different time points according to the endogenous life cycle of *A. abstrusus* in the cat. The efficacy in preventing the onset of clinical signs was not evaluated as none of the experimentally infected cats showed respiratory distress or signs during the study [110]. Then, in two studies of naturally infected cats, the same formulation showed an efficacy of 90.5–99% against aelurostrongylosis based on reduction of larval shedding. All animals in these field studies, though limited in number, recovered from pretreatment clinical signs [18,111]. To date, this parasiticide is used for treatment of adult *A. abstrusus* with one or two (at monthly interval) administrations and for preventive administration against L3 and L4.

#### 4.1.3. Novel Acquisitions and Open Perspectives

Very recently, an experimental study has demonstrated the long-term preventative efficacy against cat aelurostrongylosis of a single dose of a topical solution containing moxidectin at the minimum recommended dose of 2 mg/kg (and the isoxazoline fluralaner). This laboratory trial showed that this product is 100% and 99.66% efficacious after application 8 or 4 weeks and 12 weeks prior to experimental infection, respectively. Though generated in a single study, these data indicate that 2% moxidectin is powerful in inhibiting the establishment of adult *A. abstrusus* and preventing lung damage and larval excretion, thus meaning that a single treatment has the potential to prevent cat aelurostrongylosis for at least 12 weeks [112]. These promising data need further experimental substantiation, and it would be insightful to confirm such preventative success in cats living in enzootic regions under natural conditions. Analogously, the promising data achieved for selamectin and milbemycin oxime (Section 4.1.1), though few and limited, call for new investigations for the treatment and prevention of cat aelurostrongylosis in a large cohort of cats with these macrolactones. 

### 4.2. Troglostrongylosis

Knowledge on treatment and control options for *T. brevior* is still incipient. To date, only one product containing the macrolactone eprinomectin is used to treat and control cat troglostrongylosis, but promising data have been generated for other molecules. 

#### 4.2.1. Single Clinical Cases

A case of infection attributed to *T. subcrenatus* was unsuccessfully treated with oral fenbendazole and the animal died despite this protocol [59]. Since then, *T. subcrenatus* has never been found again, thus no further efficacy data are available. 

The first known administration of 1% moxidectin topical treatment in a cat with troglostrongylosis due to *T. brevior* appeared to be unsuccessful, probably due to the severe clinical picture and and extensive lung damage already present at the time when the cat was presented and also due to only a single application [59]. Another kitten infected with *T. brevior* was copromicroscopically and biomolecularly negative from one week to three months following a single administration of the same topical product. A complete recovery from severe cardiorespiratory signs, however, did not occur, due to the extensive pulmonary damage already present before initial treatment [41].

Again, single administration of 1% topical moxidectin showed 100% efficacy in stopping larval output in two kittens with troglostrongylosis. Additionally, one of these kittens recovered clinically within two weeks after the dose, while the second displayed clinical signs and radiographic findings for a longer period before fully recovering, likely due to an initial condition that was clinically more severe [26]. In the latter study, the product was administered twice, two weeks apart, to achieve the elimination of larvae and the recovery from clinical signs within one month in three cats coinfected by *T. brevior* and *A. abstrusus* [26]. Overall, it seems that the efficacy of 1% moxidectin vs. *T. brevior* depends on the severity of lung damages at the time of the therapy, the time of administration and the presence of mixed infections. 

Single reports have indicated a variable efficacy of 2 mg/kg milbemycine oxime in cats belonging to the same litter and infected by *A. abstrusus* and *T. brevior* (Section 4.1.1). In these cases, a single administration appeared to be effective in one cat, while in another its efficacy against *T. brevior* appeared lower than against *A. abstrusus* [42]. No extensive data are available on the usefulness of this molecule in cats with monospecific infections by *T. brevior*, as there is only one described clinical case where a single administration of milbemycin oxime was efficacious in stopping larval shedding and ensuring a clinical recovery [26].

The first pieces of sound evidence for the potential use of spot-on emodepside against *T. brevior* were published a few years ago, in cats with mixed respiratory parasitoses [90]. The formulation guaranteed the recovery from respiratory signs after one or two administrations and, moreover, larval (*T. brevior* and *A. abstrusus*) and egg (*C. aerophila*) shedding stopped 2–4 weeks after treatment [90]. A ≤2-month-old kitten with natural troglostrongylosis received two off label administrations of emodepside spot-on at 2 week intervals by a veterinary practitioner and recovered parasitologically and clinically with no adverse events [60]. 

#### 4.2.2. Labelled Formulation 

At present, only the topical parasiticide containing eprinomectin (Section 4.1.2) is used for *T. brevior* infection. A first study showed its efficacy, based on larvae counts, in treating *T. brevior* in monospecific and mixed infections with *A. abstrusus* in naturally infected cats, though in a limited number of animals [111]. Later on, data from a multicentric field survey on a higher number of animals confirmed the same 100% efficacy of a single dose of spot-on eprinomectin in eliminating *T. brevior* L1 output and curing clinical signs [18]. Laboratory results have confirmed the excellent efficacy of topical eprinomectin against this lungworm—i.e., a single administration proved to be 100 % efficacious against both developing larval and adult *T. brevior* in experimentally infected animals. The effectiveness of this parasiticide was shown by the analysis of impairment or damage to the respiratory and immune systems, which are prevented when treatment is administered during the prepatent period or are improved within three weeks of treatment of parasitized cats [113]. To date, the product is used to treat L4 and adult *T. brevior*. 

#### 4.2.3. Novel Acquisitions and Future Perspectives

Recent clinical trials have shown the potential efficacy of moxidectin and emodepside in the therapy of cat troglostrongylosis. 

A multicentre trial conducted under natural conditions proved the 100% efficacy of 1% moxidectin in eliminating *T. brevior* L1 shedding after a single treatment. As the study cats had no severe clinical signs, this study calls for further investigations to confirm the efficacy of 1% moxidectin in animals with moderate to severe troglostrongylosis and determine if one or more administrations are required to achieve a clinical recovery [114].

A pilot study has recently proved that a topical product containing emodepside has a high effectiveness after one (87.5%) or two (100%) administrations two weeks apart in terms of reducing larval shedding in naturally infected cats. This study provided also a first evaluation of the ability of this product in the clinical recovery of cats with troglostrongylosis, via the comparison of clinical scores and evaluation grids for respiratory distress and other signs pre- and post-treatment. Importantly, the vast majority of cats with clinical signs recovered after the first dose treatment [115].

Based on this very promising knowledge, other studies are advocated to confirm the therapeutic efficacy of moxidectin and emodepside against *T. brevior* in a larger number of cats under laboratory and natural conditions. Some information is missing, such as the number of administrations and dose intervals required for moxidectin and emodepside to attain a constant 100% treatment efficacy, especially in cats with severe clinical conditions, high parasite loads or mixed infections with *A. abstrusus* and/or *C. aerophila*. 

New studies enrolling animals with evident clinical signs and radiographic lesions are necessary to corroborate the efficacy of moxidectin and emodepside in clinical settings. In fact, there is no doubt on the effectiveness of these molecules in stopping larval output in cases of subclinical to mild infections [114,115]. It remains to be seen what the best protocol schemes (e.g., number of doses and interval between administrations) are to guarantee the recovery of cats with moderate to severe troglostrongylosis in terms of clinical signs and pulmonary damages.

To date, only one product is licensed to prevent troglostrongylosis (see above, topical eprinomectin). The possible efficacy of 1% moxidectin in preventing *T. brevior* has been suggested by a field trial which proved that cats at risk of troglostrongylosis do not acquire the infection when they receive the monthly chemopreventive scheme for *Dirofilaria immitis* [116]. Further trials evaluating the efficacy and duration of this molecule in the chemoprevention of troglostrongylosis are of high interest because (i) regular administration of topical moxidectin can induce sustained steady state plasma concentration [117], (ii) one product containing 1% moxidectin is already used for preventing aelurostrongylosis [109], (iii) a formulation containing 2% moxidectin has proven to be highly effective against *A. abstrusus* for 12 weeks after a single administration [112] and iv) troglostrongylosis may have a fatal outcome. 

The life-threatening potential of *T. brevior* renders the investigations on the efficacy and safety of anthelmintics in young kittens and pregnant/lactating queens essential. According to drug licenses, the products containing moxidectin, emodepside or eprinomectin can be safely administered to kittens of 9, 8, and 7 weeks (minimum) of age, respectively. Successful treatment of kittens younger than 8 weeks with emodepside spot-on formulation with no adverse events [60,90] highlight the merits of evaluating the safety of this molecule in treating troglostrongylosis in very young animals. Emodepside can be used during pregnancy and lactation and it is effective in preventing lactogenic transmission of *Toxocara cati* [108]. Accordingly, an investigation of the chemopreventative efficacy of emodepside for interruption of *T. brevior* vertical transmission to the offspring of queens is recommended.

Although no efficacy data on *T. brevior* are known for milbemycin oxime and selamectin, the characteristics of these molecules call for further trials. In fact, it is worth noting that milbemycin oxime and selamectin can be used in kittens by 6 and 8 weeks of age, respectively, and both are considered safe in pregnant and lactating queens.

### 4.3. Capillariosis

Knowledge available for successfully treating cats infected by *C. aerophila* is little, most likely because this pathogen has been neglected for a long time. To date, there has only been one parasiticide (1% spot-on moxidectin) used for treating pulmonary capillariosis in cats.

#### 4.3.1. Single Clinical Cases

Injectable or oral levamisole and off label abamectin have shown a certain efficacy at variable dosages and number of administrations in single clinical cases. Three doses of injectable levamisole (7.5 mg/kg), of which the first two were administered 24 h apart and the third 14 days later, were reported to be effective in an old case report, with reduction in clinical signs and cessation of egg shedding [118]. Another anecdotal protocol using three treatment cycles of oral levamisole at a dose of 5 mg/kg for 5 consecutive days, with a 9-day interval between each cycle, showed some efficacy in the treatment of a naturally infected cat [119]. In this latter case, clinical signs disappeared at day 30 after the first administration and the cat was negative at copromicroscopy at day 40. Nevertheless, the results of this latter report should be interpreted with caution as the egg shedding was evaluated using an inappropriate technique—i.e., the Baermann test. 

In a further case report, two doses of 300 μg/kg abamectin administered subcutaneously 2 weeks apart produced a clinical and parasitological recovery [120]. 

#### 4.3.2. One Labelled Formulation

The same spot-on formulation containing 1% moxidectin, which is used for treatment and prevention of aelurostrongylosis, is claimed to treat *C. aerophila* in cats. An early study demonstrated 99.79% efficacy to halt egg shedding in cats with natural infection. Additionally, all treated cats with pretreatment respiratory signs were clinically healthy at the end of the study [44]. More recently, this efficacy was confirmed by a field trial which proved that a single administration of 1% moxidectin at the recommended dose is 100% efficacious in reducing *C. aerophila* egg output and in clinically curing the vast majority of infected cats [121]. This formulation is now used for treating adult *C. aerophila* in cats with a single administration.

#### 4.3.3. Novel Acquisitions and Future Perspectives

A study showed the efficacy of the topical combination containing eprinomectin against helminths in domestic cats, including 99.6% ability in reducing counts of *Capillaria* spp. eggs after treatment. Though no identification of species of these eggs was provided, it was assumed that the vast majority was *C. aerophila* [16]. Later, a controlled study in naturally infected cats that were necropsied after a single treatment with the above product proved 93.5–99.1% and 82.4% efficacious in reducing the output of *C. aerophila* ova and worm counts, respectively [17]. A recent multicentered field study has proved that the topical parasiticides containing 2% moxidectin (plus fluralaner) and 2.1% emodepside (plus praziquantel) have the potential to eliminate (efficacy 100%) *Capillaria* spp. eggs from the feces of naturally infected cats [122]. Again, these eggs were not identified at the species level; thus, precise values for *C. aerophila* are unavailable for these products. However, in the clinical case of a cat with mixed clinical capillariosis and troglostrongylosis (see Section 4.2.1) emodepside was efficacious in stopping shedding of *C. aerophila* eggs from one to four weeks (i.e., the last fecal examination performed) post-treatment. Additionally, the cat showed a clinical improvement one week after treatment and displayed no clinical signs at 2, 3 and 4 weeks follow-up [90]. 

As only one formulation, containing 1% moxidectin, is currently licensed for treating *C. aerophila* in cats, the efficacy of the same molecule, contained in a higher amount (i.e., 2% moxidectin) with fluralaner, was confirmed and the promising activity of eprinomectin and emodepside have already been shown in field trials. 

No data are available on the efficacy of milbemycin oxime and selamectin in the treatment of respiratory capillariosis of cats. Thus, the first pilot trials, even in small clinical cohorts of cases, are advocated here. 

### 4.4. Clinical Efficacy 

Severity of lung damage, parasitic burden, nematode(s) involved, cat age, number of dose administrations and concomitant infections could represent key drivers having an impact on the efficacy of anthelmintics in treating feline lungworms. Accordingly, comparative clinical studies in naturally infected cats in single and mixed infections are important in order to elucidate the factors impacting complete parasitological, clinical and radiographic recovery. At the moment, this important knowledge is mainly limited to the use of emodepside and moxidectin in cats infected by *A. abstrusus*, while less data is available for other molecules and for *T. brevior* and *C. aerophila*.

Spot-on 1% moxidectin proved to have a high clinical efficacy in cats with natural aelurostrongylosis. In a clinical study, 12 cats with a monospecific infection by *A. abstrusus* were treated with 1% moxidectin, and after a single administration, all but one of them recovered clinically within two weeks of treatment, while radiographic signs disappearing in seven of them [26]. At 28 days post-treatment, all cats were healthy and radiographic alterations were absent in ten of them [26].

In the same case series [26], a single dose of a spot-on formulation containing 2.1% emodepside resulted in full clinical recovery and remission of the radiographic alterations in a cat coinfected with *A. abstrusus* and *T. brevior* when evaluated 14 days post-treatment. Afterwards, a controlled field study showed high clinical efficacy with two administrations of emodepside, repeated 2 weeks later, in ensuring complete remission of clinical and radiographic signs in eight cats with natural *A. abstrusus* monospecific infections within 10 weeks of the first administration [97]. In this latter study, abnormal breath sounds (e.g., crackles) disappeared in around half of the infected cats 14 days after the first administration, while two weeks after the second administration, all study cats did not display clinical signs, with statistically significant differences when compared to the control group [97]. Radiographic alterations disappeared in 3/8 cats and 5/8 after the first and the second administration, respectively. None of the treated cats displayed any lesion observable at X-ray imaging at 42 days after the second dose of emodepside [97].

Other molecules showed clinical efficacy in treating aelurostrongylosis, although in a lower number of documented cases. For instance, 50 mg/kg oral fenbendazole administered for three consecutive days led to clinical and radiological resolutions after 14 days in three *A. abstrusus*-positive cats; a single dose of 2 mg/kg oral mylbemicine oxime was used in a cat coinfected by *A. abstrusus* and *T. brevior* [26]. Promising data are available for selamectin as two doses administered 24 days apart in 10 cats ensured the remission of clinical signs 37 days after the second administration [105].

Data on the efficacy of parasiticides in treating troglostrongylosis in terms of clinical signs and pulmonary alterations are still meagre. Information available on the only product thus far licensed vs. *T. brevior* mostly comes from post-treatment evaluations of worm counts and/or larval shedding, while its activity on clinical signs and pulmonary damage has been investigated in a small number of cats or in experimental settings [18,111,113].

While 1% moxidectin proved to be highly effective in stopping larval shedding in cats with subclinical troglostrongylosis even after one dose [114], current data suggest that cats with moderate to severe signs would require timely treatments and sometimes more than one administration. In the clinical comparative study cited above for aelurostrongylosis, other than the reduction in larval shedding, 1% topical moxidectin ensured complete clinical recovery within two and six weeks, respectively, in two cats infected with *T. brevior* and radiographic recovery within 4–6 weeks in three cats coinfected by *T. brevior* and *A. abstrusus* [26].

The first pilot trial aimed at evaluating the efficacy of emodepside against *T. brevior* proved that this molecule is efficacious in curing clinical signs in naturally affected cats [115]. Data from this study showed that cats may start to improve clinically 2–4 weeks post-treatment and that they become healthy 6 weeks after dosing. Nevertheless, no comparative radiographic analysis of pulmonary alterations was performed pre- and post-treatment in this study. Cats infected by lungworms may still have lung changes, although clinical signs disappear and larval shedding stops after therapy [25,48,51]. Thus, further clinical studies on large numbers of cats are warranted to understand in which terms a factual improvement of pulmonary damages takes place after treatment with emodepside. This is particularly important, as single case reports showed that one dose of this parasiticide was effective in interrupting larval output with the clinical and radiological recovery in two kittens with *T. brevior* and either *A. abstrusus* or *C. aerophila* two weeks post administration [90].

Clinical deterioration may be observed in a few cats soon after treatment against *T. brevior* due to the death of parasites with subsequent inflammatory response and appearance of acute clinical signs [115]. To the best of the authors’ knowledge, a similar occurrence for *A. abstrusus* has been described only in a single case of a 5-month-old kitten from the USA [58], but it cannot be excluded that this could occur more frequently. This suggests that a concomitant administration of anti-inflammatory drugs such as prednisolone (1–2 mg/kg) or dexamethasone (0.1–0.2 mg/kg) [98] could be of benefit in cats severely infected with *A. abstrusus* or *T. brevior*. Indeed, veterinary practitioners often suggest this therapeutic approach in feline respiratory parasitoses [25,123] even though no trials have been conducted to understand whether the association of anti-inflammatory drugs to anthelmintic treatments in cats with lungworms may improve and/or accelerate a clinical remission after treatment.

Clinical data on the effectiveness of anthelmintics in assuring parasitological, clinical, and radiographic remission in cats infected by *C. aerophila* are also poor. After promising data were gathered ten years ago from a limited number of cats [44], a more recent trial has proved that a single administration of 1% moxidectin may provide a marked, though sometimes incomplete, clinical improvement within a ~10-day period postdosing [121]. Preliminary evidence indicates that emodepside and eprinomectin have the potential to treat clinical lung capillariosis but sound data are still necessary to confirm this evidence in large cohorts of animals [26,42]. 

## 5. Concluding Remarks

The last analysis and review of the literature available on the practical and clinical importance of respiratory nematodes was published in 2016 [4]. In the past five years, more knowledge on feline respiratory parasites has been disseminated through case reports, epizootiological surveys, comparative clinical studies and efficacy treatment and control trials. 

It is ultimately confirmed that the “cat lungworm” *A. abstrusus* remains the most important respiratory nematode of cats, but *T. brevior* and *C. aerophila* play an increasingly relevant role in feline clinical parasitology. At the same time, novel data have proven that other nematodes, occasionally recorded in the lungs of cats, i.e., *O. rostratus*, *T. subcrenatus* and *A. chabaudi*, play negligible to secondary roles in cat parasitology. Specifically, *O. rostratus* very rarely infects domestic cats and its pathogenic potential is unknown, the species identity of *T. subcrenatus* as a valid taxon is questionable, and no patent or clinical infection by the pulmonary artery nematode, *A*. *chabaudi,* has been described in domestic cats thus far [1]. For these reasons, these three nematodes have not been included in the present review. 

Clinical signs in cats infected by respiratory nematodes overlap each other, and a clinical suspicion of a given species is almost impossible due to the high risk of mixed infections in enzootic areas. However, in some particular cases identification of a more plausible nematode rather than another is possible—e.g., *A. abstrusus* in chronic infections of adult cats, *T. brevior* in kittens or young animals with severe signs, and *C. aerophila* in cats living where foxes are also present. Regardless of the species involved, any respiratory sign in cats should require that veterinarians and clinicians perform appropriate diagnostic tests. Additionally, routine detection of cats for lungworms is of importance as healthy cats apparently may already have pulmonary damage detectable upon radiographic examination. This is also true for all cats which should undergo any surgery or receive general anesthesia given that there is a factual hazardous risk associated with these parasites [124].

Although cats infected with *A. abstrusus* and/or *T. brevior* may show overlapping radiographic alterations, novel knowledge is necessary to understand what alterations occur in cats with either troglostrongylosis or lung capillariosis alone. When presented with respiratory sings, cats are more often subjected to the Baermann’s method rather than to a fecal flotation. Therefore, it should be understood which radiographical changes may indicate that veterinarians should perform a fecal flotation instead of a sole Baermann examination in order to detect a possible *C. aerophila* infection. 

Further investigations are warranted to understand whether these parasites induce nonrespiratory clinical signs. It has been shown that these respiratory nematodes could cause cardiovascular damages more often than has been previously thought, and accordingly, timely diagnosis and treatment are crucial to avoid severe and/or irreversible lesions to the lung parenchyma and vessels, especially in young animals. In this view, studies with longer follow-up controls are needed to understand if PH can be factually characterized as “irreversible” or if the resolution of cardiac abnormalities requires more time than three months, as in existing clinical records [41]. 

Known clinical cases indicate that an echocardiography examination could be useful in the clinical workup of cats infected by *A. abstrusus* and *T. brevior* to individuate possible PH and appropriate treatments and follow-up. Thus, *A. abstrusus* and *T. brevior* must be included in the differential diagnosis when heart murmur, PH and/or right-sided congestive failure are detected, especially in kittens and young cats. Large cohorts of infected animals should be examined to understand the factual occurrence of PH in cats infected by these parasites in relation to their clinical picture and thoracic alterations. 

Studies are warranted to investigate whether similar cardiopulmonary alterations also occur when cats harbor *C. aerophila*. 

Advanced imaging methods such as CT scans are not routinely used in respiratory medicine of cats. The usefulness of these methods to better understand pulmonary lesions is unquestionable and they are particularly promising for further investigations aiming at showing the long-term clinical efficacy of parasiticides. This advanced approach could be used for thorough analysis of respiratory lesions that are not detected by radiography, especially in terms of post-treatment follow-up. Further studies are advocated to investigate the usefulness and the significance of CT scans in cats infected by *T. brevior* and/or *C. aerophila*. 

Data recently arisen on the morphometrics of lungworm diagnostic stages has put in question the factual utility of larval detection and identification in laboratory and clinical settings for a definitive diagnosis of infection. Information gathered in previous years allow any skillful microscopist to reliably identify L1 of *A. abstrusus* and *T. brevior* and eggs of *C. aerophila*. When required, genetic assays may be used to overcome the inherent drawbacks of conventional methods. Serological assays are indeed promising, though a certain refinement is necessary before a definitive validation for use in daily clinical practice (e.g., validation of rapid in-clinic tests). 

Until a few years ago only a few formulations were licensed for aelurostrongylosis. In more recent years, the primary importance of these parasites in feline clinical parasitology was acknowledged and, accordingly, many laboratory and field trials have been carried out to evaluate the efficacy of anthelmintics in treatment and control of the infections caused by *A. abstrusus*, *T. brevior* and *C. aerophila*. To date, spot-on moxidectin (in 1% solution) and eprinomectin are both used to treat and prevent aelurostrongylosis, and also to treat either capillariosis or troglostrongylosis, respectively. Topical emodepside and eprinomectin are licensed to treat aelurostrongylosis or prevent troglostrongylosis, respectively (Table 2). There are also sound indications that further studies in laboratory and clinical conditions would increase the number of treatment and chemoprevention label claims. In fact, various molecules have shown promising activity to treat parasitic stages and species for which they do not have (yet) the marketing authorization (Table 2). At the moment, only 1% moxidectin (vs. *A. abstrusus*) and eprinomectin (vs. *A. abstrusus* and *T. brevior*) are licensed to kill larval lungworms in the cats with monthly administrations. More data are warranted to explore the potential of parasiticides in field control of these infections through chemopreventative regimens. In addition, a specific season for targeted chemoprevention of lungworms is difficult to identify. Therefore, recommendations for chemoprevention would consider each local scenario and take into account that cats may be at risk of infection throughout the year. In this view, the recent evidence of 2% topical moxidectin in preventing cat aelurostrongylosis for about 3 months after one dose [112] would be particularly important to protect animals all year round with administrations required less frequently than once a month. Comparative clinical trials are advocated to understand which molecule/s is/are the most effective not only in killing parasite adult and larval stages in cats with pre-existing infections but also in assuring a clinical remission of signs and radiographic alterations. In fact, gaps have yet to be filled to improve understanding the efficacy of anthelminitics to treat diseases caused by feline respiratory nematodes as, to date, most data are entirely based on the reduction in larval or egg output and/or worm counts.

It is important to understand what factors may influence the effectiveness of various parasiticides—e.g., number of doses, interval doses parasitic burden, single or mixed infections, clinical scores of signs and thoracic alterations—and if there is an identifiable and measurable impact of these drivers on each molecule or if there are compounds with higher and/or quicker activities to ensure the clinical remission of cats regardless the abovementioned factors. For instance, this could be interesting for 2% moxidectin, which recently proved to be effective in the prevention of aelurostrongylosis in experimental settings [112]. Given that single or repeated administrations of 1% moxidectin showed varying degrees of efficacy against *A. abstrusus*, *T. brevior* and *C. aerophila*, studies are warranted to investigate if higher concentrations of the molecule have an influence on the number of administrations needed to ensure faster interruption of parasite shedding and remission of clinical and radiographic alterations. In more detail, 1% topical moxidectin (Section 4.1.2) is already used for treating aelurostrongylosis, with up to three monthly doses at a minimum recommended dose of 1 mg/kg. It could be interesting to understand whether the same efficacy could be achieved with fewer administrations of the product marketed at the minimum recommended dose of 2 mg/kg. New data supporting the preliminary knowledge on the preventative efficacy of this product are also expected. Efficacy trials with marketed formulations containing moxidectin at different concentrations are highly desirable in the case of troglostrongylosis and capillariosis. These should investigate if the amount of the macrolactone has an impact not only on reducing parasite output, but also on the recovery from clinical signs and pulmonary lesions. For both treatment and prevention protocols, it would be worthwhile to unveil their efficacy in terms of number of applications and dose intervals. More investigations on the clinical efficacy are advocated for emodepside, moxidectin and eprinomectin, for which data are mostly available only for *A. abstrusus* and emodepside and moxidectin 1%. No preliminary data on the clinical effectiveness of milbemycin oxime and selamectin against *A. abstrusus*, *T. brevior* and *C. aerophila* are available, thus basic studies are here called for. 

It is worth mentioning that these parasites may affect wildlife, even with severe and life-threatening clinical signs, e.g., pulmonary lesions, and have developed reproduction strategies which favor their transmission patterns [1,125,126,127]. More data should be generated on the clinical impact of lungworms on endangered felids and on possible therapeutic options [125,126].

In conclusion, the amount of studies conducted in the past few years on felid lungworms have provided novel and important data in clinical settings. At the same time, new perspectives have been introduced due to recent information and new studies need to be conducted in the near future for the implementation of diagnostic and control methods.

## Figures and Tables

**Table 1:** Morphometric (in μm) and morphologic characteristics of first-stage larvae (L1) of *Aelurostrongylus abstrusus* and *Troglostrongylus brevior*. The length ranges in the table are reported in the references cited.

Parasite	Anterior End	Posterior End	Length/Reference
*Aelurostrongylus abstrusus*	- plateau-like - terminal oral opening	- ventral kink - deep dorsal and ventral incisures - knob-like appendages	300–400 ± 21.3 / [5]
			360–415 (399.1 ± 11.3) / [74]
			210.4–495.1 / [18]
*Troglostrongylus brevior*	- apical prominence - subterminal oral opening	- shallow/absent ventral kink - dorsal incisure present - shallow ventral incisure - slender terminal appendix	300–521 / [5]
			300–357 (338.8 ± 15.6) / [74]
			203.2–382.2 / [18]

**Table 2:** Specific anthelmintic ingredients in parasiticide formulations for whom investigators/companies have claimed treatment (T) and/or prevention (P) success of infections caused by *Aelurostrongylus abstrusus* (Aa), *Troglostrongylus brevior* (Tb) and *Capillaria aerophila* (Ca), and molecules for which there is off label data indicating promising effectiveness against these parasites.

Molecule	Aa	Tb	Ca	Off Label	Refs
2.1% Emodepside	Yes (T)	No	No	Tb (T) Ca (T)	[60,90,107,108,122]
1% Moxidectin	Yes (T/P ^1^)	No	Yes (T)	Aa (P ^4^) Tb (T/P ^5^)	[106,109,112,114,121]
0.4% Eprinomectin	Yes (T/P ^2^)	Yes (T/P ^3^)	No	Ca (T)	[18,26,110,111,113]
Milbemycin oxime ^6^	No	No	No	Aa Tb (T) Ca (T)	[26,42]
6% Selamectin	No	No	No	Aa (T)	[22,104,105]

^1^ Efficacy against third- and fourth-stage larvae and adult stage of a marketed topical solution containing 1% moxidectin; ^2^ efficacy against third- and fourth-stage larvae; ^3^ efficacy against fourth-stage larvae; ^4^ a marketed topical solution containing 2% moxidectin with proved efficacy against early larval stages in one experimental trial [112]; ^5^ a field trial has indirectly suggested the efficacy of a topical solution containing 1% moxidectin in preventing troglostrongylosis in cats living in enzootic regions [116]; ^6^ 2 mg/kg.

## Data Availability

No new data were created for this review article.
